# Genetic Relationship of Fall Armyworm (*Spodoptera frugiperda*) Populations That Invaded Africa and Asia

**DOI:** 10.3390/insects12050439

**Published:** 2021-05-12

**Authors:** Rajendra Acharya, Ashraf Akintayo Akintola, Matabaro Joseph Malekera, Patrick Kamulegeya, Keneth Benedictor Nyakunga, Munyaradzi Kennedy Mutimbu, Yam Kumar Shrestha, Jahan S. M. Hemayet, Trinh Xuan Hoat, Hang Thi Dao, Jeong-Hoon Park, Iksoo Kim, Moon Nam, Sung-Jin Lee, Sang-Mok Kim, Hwal-Su Hwang, Kyeong-Yeoll Lee

**Affiliations:** 1 Department of Applied Biosciences, College of Agriculture and Life Sciences, Kyungpook National University, Daegu 41566, Korea; racharya2048@gmail.com (R.A.); ashraf.akintola@gmail.com (A.A.A.); jmatabaro@live.com (M.J.M.); bgtwo2@naver.com (H.-S.H.); 2 Department of Zoology, Faculty of Life Sciences, University of Ilorin, Ilorin 240212, Nigeria; 3 Department of Plants Protection, Ministry of Agriculture, Kinshasa 8722, Democratic Republic of the Congo; 4 Ministry of Agriculture Animal Industry and Fisheries, Entebbe P.O. Box 102, Uganda; pkamulegeya@yahoo.com; 5 National Biological Control, Kibaha 30031, Tanzania; kennybenny83@yahoo.com; 6 Department of Applied Sciences, Mutare Polytechnic College, Mutare P.O. Box 640, Zimbabwe; mmutimbu@gmail.com; 7 Center for Industrial Entomology, Hariharbhawan, Lalitpur 44700, Nepal; ykshrestha@gmail.com; 8 Department of Entomology, Patuakhali Science and Technology University, Dumki, Patuakhali 8602, Bangladesh; hemayet_pstu@yahoo.com; 9 Plant Protection Research Institute, Ha Noi 04, Vietnam; trinhxuanhoat.ppri@mard.gov.vn (T.X.H.); daothihang@hotmail.com (H.T.D.); 10 Jejudo Agricultural Research and Extension Services, Jejudo 63556, Korea; pjh0221@korea.kr; 11 College of Agriculture and Life Sciences, Chonnam National University, Gwangju 61186, Korea; ikkim81@chonnam.ac.kr; 12 Xenotype, Daejeon 34912, Korea; moonlit51@xenotype.co.kr; 13 Plant Quarantine Technology Center, Animal and Plant Quarantine Agency, Gimcheon 39660, Korea; mycomania21@korea.kr (S.-J.L.); supraorder@korea.kr (S.-M.K.); 14 Institute of Plant Medicine, Kyungpook National University, Daegu 41566, Korea

**Keywords:** *Spodoptera frugiperda*, invasive pest, corn, invasion, *COI*, *Tpi* gene

## Abstract

**Simple Summary:**

Since 2016, the fall armyworm, an important economic pest native to tropical and subtropical regions of the Western Hemisphere, has invaded Africa and further spread rapidly into most Asian countries. The fall armyworm is highly polyphagous, but two of its major strains, the corn and the rice strains, cause severe damage in the Western Hemisphere. However, the invaded populations in Africa and Asia mostly infested the corn fields. Studies on the genetic identity of the species using two molecular markers, one nuclear gene and one mitochondrial gene, showed that the major genetic group is a heterogeneous hybrid of males from the corn strain and females from the rice strain. Moreover, a minor group of homogenous individuals from the corn strain but no homogenous individuals from the rice strain were also detected. A geographic distribution analysis at the subpopulation level indicated similar genetic diversity in Africa and Asia, suggesting fall armyworm in Africa spread into Asia without significant genetic change.

**Abstract:**

The fall armyworm, *Spodoptera frugiperda*, is an important agricultural pest native to tropical and subtropical regions of the Western Hemisphere, and has invaded Africa and further spread into most countries of Asia within two years. Here, we analyzed the genetic variation of invaded populations by comparing the nucleotide sequences of two genes: the nuclear Z-chromosome linked gene *triose phosphate isomerase* (*Tpi*) and the mitochondrial gene *cytochrome oxidase subunit I* (*COI*) of 27 specimens collected in Africa (DR Congo, Tanzania, Uganda, and Zimbabwe) and Asia (Bangladesh, Korea, Nepal, and Vietnam). The results revealed that 25 specimens were from a heterogeneous hybrid (*Tpi*-corn strain and *COI*-rice strain; *Tpi*-C/*COI*-R) of the corn strain male and rice strain female, but two specimens were from a homogenous corn strain (*Tpi*-corn strain and *COI*-corn strain; *Tpi*-C/*COI*-C). The further analysis of the fourth exon and the fourth intron sequences of the *Tpi* gene identified at least four subgroups of the corn strain. These four genetic subgroups were identified in Africa and Asia, suggesting no significant genetic change due to the rapid migration within two years. Our study provides essential information for understanding the genetic diversity of fall armyworm in new habitats.

## 1. Introduction

The fall armyworm (FAW) *Spodoptera frugiperda* (J. E. Smith, 1797) (Lepidoptera: Noctuidae) is an important agricultural pest native in tropical and subtropical regions of the Western Hemisphere [1]. Due to the lack of diapause mechanism, FAW cannot overwinter in the northern areas over Florida and Texas of the United States, but they can disperse across thousands of kilometers into the north in the growing season [2]. In 2016, its invasion into Western Africa was first reported and it rapidly spread into most Sub-Saharan Africa countries [3,4,5,6]. In 2018–2019, the invasion into India was firstly reported and further spread into most Asia-Pacific countries, including Korea, Japan, and Australia, within an year [7,8,9,10,11,12,13,14]. The enormous migratory power of the FAW is a severe threat to new habitats in Africa and Asia and poses as a significant concern related to the potential economic damage of crop plants [15,16,17].

The FAW is a polyphagous species, consuming at least 353 species of plants, and it is a significant pest of corn, rice, and forage grasses [18,19]. Pashley et al. [20] showed at least two host plant strains in the southeastern United States: one of them feeding on corn, cotton, and sorghum (corn strain, C-strain) and the other feeding on rice and various pasture grasses, preferentially (rice strain; R-strain) [18,21,22]. The two FAW strains are morphologically indistinguishable and are distributed in sympatric patterns [23]. Further studies identified their different genetic characteristics in mating behaviors and zygotic reproductive incompatibility [24,25], pheromone composition [26], and differential susceptibility in xenobiotics [27].

Molecular markers can be used to diagnose the genetic identity of each strain of FAW [28,29]. Polymorphic variation of mitochondrial *cytochrome oxidase subunit I* (*COI*) gene sequence was identified between C- and R- strains but was not always consistent with host plant preference [30,31]. For example, some populations collected from the cornfields possess an R-strain marker in the *COI* gene. The group of Nagoshi and collaborators developed another genetic marker using a nuclear *triosephosphate isomerase* (*Tpi*) gene linked with Z-chromosome [32,33]. Therefore, the *Tpi* gene is hemizygous in females (ZW), whereas in males it is either homozygous or heterozygous (ZZ) [32]. Two genotypes, *Tpi*-C and *Tpi*-R, were identified on different host plants in the Western Hemisphere [31,32]. The group of Nagoshi and collaborators found that significant corn field populations are a hybrid (*Tpi*-C/*COI*-R) that possesses a nuclear *Tpi*-C marker but a mitochondrial *COI*-R marker. This finding indicates that the host plant preference of the hybrid is associated with the nuclear *Tpi* marker rather than the mitochondrial *COI* marker [34]. Therefore, it suggests that the *Tpi* gene is a suitable molecular marker compared with the *COI* gene to identify the FAW genetic characteristics associated with the host plant preference of the species.

Here, we assessed the genetic variation of FAW specimens collected from eight African and Asian countries based on a *Tpi* gene and compared with their variation of *COI* gene markers. Moreover, we discussed the relationship between genetic diversity and the potential population dynamic of FAW populations that invaded the new African and Asian habitats.

## 2. Materials and Methods

### 2.1. Collection

**Figure 1:**
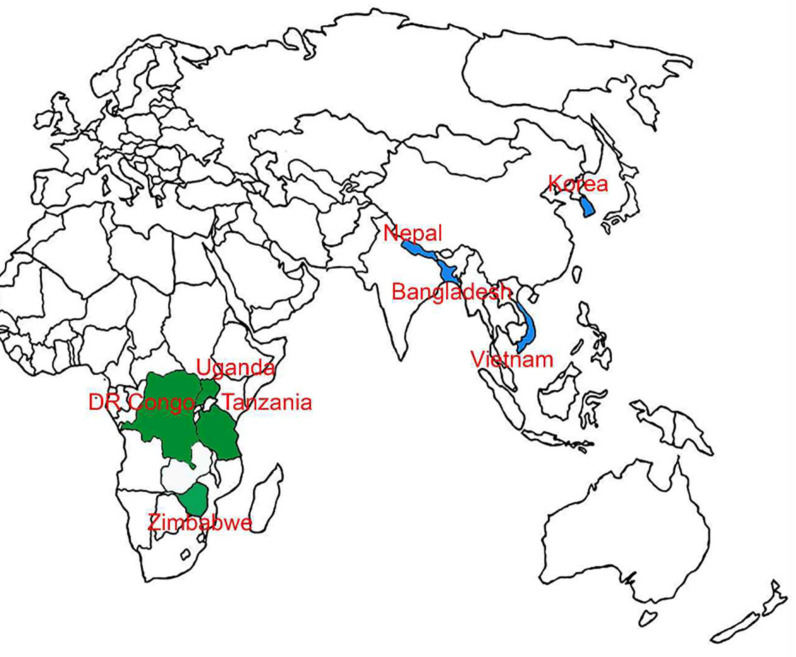
Map showing the collection sites of *Spodoptera frugiperda* specimens in the different African and Asian countries.

The FAW larvae were collected from corn fields (*Zea mays* L.) in Gyeongsan, Gyeongbuk Province, and adult moths were caught using the sex pheromone traps (GreenAgrotech, Gyeongsan, Korea) in Jeju Island of Korea from 2019 to 2020. Other specimens were obtained as larvae and adults from corn plants at various locations in Africa (DR Congo, Tanzania, Uganda, and Zimbabwe) and Asia (Bangladesh, Korea, Nepal, and Vietnam) from October 2017 to August 2020 (Figure 1; Table 1). In the field, FAW was identified based on the morphological characteristics of larva and adults. Specimens were stored 70% ethanol. Then, the vials were stored at −20 °C until further analysis.

### 2.2. DNA Preparation

Genomic DNA was extracted from a portion of each specimen and homogenized using the pure link genomic DNA mini kit (Invitrogen, Carlsbad, CA, USA). The specimens were placed in a 1.5 mL centrifuge tube containing 180 µL of digestion buffer and 20 µL of proteinase K (50 µg/mL) and then incubated at 55 °C for 4 h. The DNA samples were extracted and purified using genomic spin columns, as described in the kit. DNA concentration was determined using a NanoPhotometer™ (Implen GmbH, Schatzbogen, Germany).

### 2.3. Polymerase Chain Reaction (PCR) Amplification

PCR was performed in a total reaction volume of 30 µL, containing 15 µL Solg^TM^ 2 × Taq PreMix (Solgent, Daejeon, Korea), 2 µL of each primer (10 pmol/µL), 3 μL of the DNA solution, and 8 μL distilled water. A partial sequence (444 bp) of the *Tpi* gene was amplified using the primer pair TPI412F (5′-CCGGACTGAAGGTTATCGCTTG-3′) and TPI1140R (5′-GCGGAAGCATTCGCTGACAACC-3′) [15], whereas the partial sequence (658 bp) of the *COI* gene was amplified using the primer pair LCO1490 (5′-GGTCAACAAATCATAAAGATATTGG-3′) and HCO2198 (5′-TAAACTTCAGGGTGACCAAAAAATCA-3′) [35]. The reaction mixtures were amplified under the following conditions: *Tpi* gene (initial denaturation at 94 °C for 1 min; followed by 33 cycles of 92 °C for 30 s, 56 °C for 45 s, and 72 °C for 45 s; and a final segment of 72 °C for 3 min], and *mt*COI (initial denaturation at 92 °C for 5 min; followed by 35 cycles 92 °C for 60 s, 55 °C for 60 s, and 72 °C for 60 s; and a final extension at 72 °C for 5 min in SimpliAmp 96-Well Thermal Cycler [Applied Biosystems, Foster City, CA, USA]). The PCR products were separated using a 1% agarose gel electrophoresis, stained with ethidium bromide solution, and visualized under ultraviolet (UV) light. The amplified PCR products were excised from the gel and purified using the Wizard^®^ PCR Preps DNA Purification System (Wizard^®^ SV Gel, Promega Co., Madison, WI, USA).

### 2.4. DNA Sequence Analysis

The purified DNA was sequenced using the BigDye^®^ Terminator Cycle Sequencing Kit and ABI Prism 3730XL DNA Analyzer (50 cm capillary) (DNA Sequencer) (Applied Biosystems, Foster City, CA, USA) at the Solgent Sequencing Facility (Solgent Co., Daejeon, Korea). The GenBank database in the National Center for Biotechnology Information (NCBI) was searched using the BLAST algorithm [36], and the nucleotide sequences were aligned using CLUSTAL W [37].

### 2.5. Phylogenetic Analysis

A phylogenetic tree for the *COI* gene was constructed using the maximum likelihood method implemented in MEGA 6.0 software [38] with reference sequences obtained in the GenBank. We used 1000 bootstrap replicates to test the robustness of each of the phylogeny with the Hasegawa–Kishnio–Yano (HKY850) model and gamma distribution rate of variation among sites [39].

### 2.6. Characterization of the Tpi and COI Gene Segments

Single nucleotide substitutions of *Tpi* and *COI* genes were used for strain diagnostic markers. The *Tpi* gene was designated by a “g” (genomic), whereas the *COI* gene was designated by an “m” (mitochondria followed by gene name, base pairs number from the predicted translational start site). In both *Tpi* and *COI* genes, we aligned our FAW specimen sequences with the previously identified NCBI sequences of C-strain and R-strain FAW in CLUSTAL W to find polymorphic nucleotides to identify both the C and R strains. Nagoshi et al. [15] reported various polymorphic nucleotides in the exon (*Tpi*-E4) and intron region (*Tpi*-I4) of the *Tpi* gene to identify the C- and R-strains. The gTpi183 (C for C-strain, T for R-strain) is used to identify C- and R-strains [15]. Both gTpi192 and gTpi198 were used to identify subgroups (*Tpi*-Ca1, *Tpi*-Ca2, and *Tpi*-Ca1/Ca2) of C-strain. In addition, various polymorphic nucleotides in *Tpi*-I4 were used to identify genetic variation of FAW.

### 2.7. Genetic Analyses

Genetic parameters, such as the number of segregating sites, haplotype numbers, haplotype diversity, nucleotide diversity, theta/site, and Tajima’s D [40], were analyzed using the DnaSP software v.5.10 [41,42]. The TCS software v.1.21 was used to generate the haplotype network [43]. We excluded the Kor-1 specimen, the hybrid of *Tpi*-Ca1/Ca2, from all genetic analyses with the *Tpi* gene.

## 3. Results

### 3.1. Analysis of the Tpi Gene Sequence

**Figure 2:**
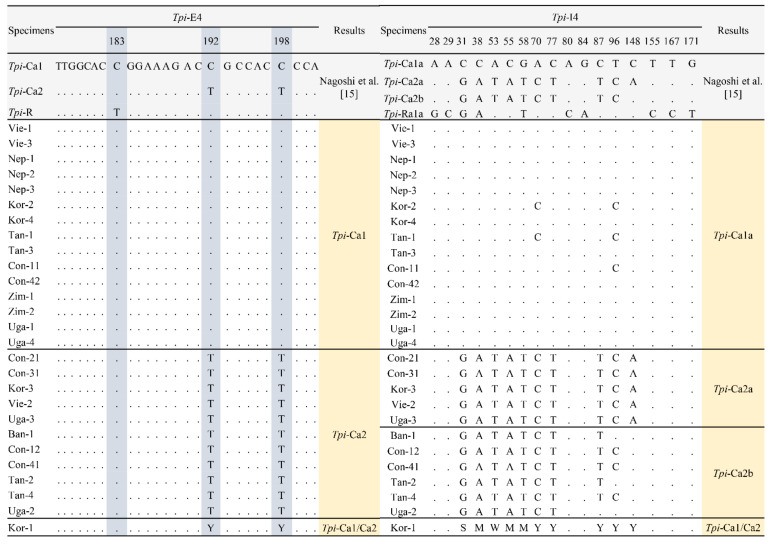
Polymorphic sites of the *Tpi* gene segments used for strain identification and haplotype diagnosis of *Spodoptera frugiperda* collected from different African and Asian countries.

The partial nucleotide sequence (444 bp), including 166 bp of the fourth exon (*Tpi*-E4) and 278 bp of the fourth intron (*Tpi*-I4) region of the *Tpi* gene, was determined from the 27 specimens of the FAW specimens collected from eight different African and Asian countries. Single nucleotide polymorphism (SNP) characteristics of the *Tpi* gene were analyzed separately in *Tpi*-E4 and *Tpi*-I4 regions (Figure 2).

In the *Tpi*-E4 region, the gTpi183 of all 27 specimens was C, but not T, which indicated that all of them were the corn strain. Furthermore, nucleotides of both the gTpi192 and the gTpi198 consisted of three different types, such as C and C, T and T, and Y (C/T) and Y (C/T), which indicates the three subgroups of corn strain, *Tpi*-Ca1, *Tpi*-Ca2, and *Tpi*-Ca1/Ca2, respectively (Figure 2). The heterozygote *Tpi*-Ca1/Ca2 specimen (Kor-1) was identified only from Jeju, Korea, in 2019.

In the *Tpi*-I4 region, among 17 polymorphic nucleotides, we found three different polymorphic sequences of *Tpi*-C in our samples but did not identified *Tpi*-R sequences reported by Nagoshi et al. [15]. Ten polymorphic nucleotides (31, 38, 53, 55, 58, 70, 77, 87, 96, and 148) were identified between *Tpi*-Ca1 and *Tpi*-Ca2 subgroups. Among them, the nucleotide 148 was distinct polymorphic nucleotide between *Tpi*-Ca2a and *Tpi*-Ca2b. In addition, nucleotide variation within subgroup was identified in some specimens of *Tpi*-Ca1 and *Tpi*-Ca2b but was not detected in *Tpi*-Ca2a. For example, in *Tpi*-Ca1 subgroup, Kor-1 and Tan-1 specimens were substituted the nucleotides 70 and 96 into “C” but Con-11 specimen was substituted only the nucleotide 96 into “C”. In *Tpi*-Ca2b, three types of variation in the nucleotides 87 and 96 were identified, for example, T and T, T and C, and C and T, respectively. Moreover, in the *Tpi*-Ca1/Ca2 heterozygote specimen, all ten polymorphic nucleotides were heterozygous into S (C/G), M (C/A), W (A/T), and Y (C/T). Therefore, our 27 specimens were classified into four subgroups as *Tpi*-Ca1a, *Tpi*-Ca2a, *Tpi*-Ca2b, and *Tpi*-Ca1/*Tpi*-Ca2.

The SNP pattern of the *Tpi* gene was analyzed according to geographic distribution (Figure 2). The results showed that each subgroup was distributed in both Africa and Asia. For example, the *Tpi*-Ca1a subgroup was identified in DR Congo (Con-11, 42), Tanzania (Tan-1, 3), Uganda (Uga-1, 4), and Zimbabwe (Zim-1, 2) in Africa as well as in Nepal (Nep-1, 2, 3), Vietnam (Vie-1, 3), and Korea (Kor-2, 4) in Asia. *Tpi*-Ca2a was identified in DR Congo (Con-21, 31) and Uganda (Uga-3) in Africa, as well as Vietnam (Vie-2) and Korea (Kor-3) in Asia, whereas *Tpi*-Ca2b was identified from DR Congo (Con-12, 41), Tanzania (Tan-2, 4) and Uganda (Uga-2) in Africa, as well as Bangladesh (Ban-1) in Asia. The *Tpi*-Ca1/*Tpi*-Ca2 was identified only in Korea (Kor-1). The results showed that each subgroup was widely distributed in both continents in a mixed pattern.

### 3.2. Analysis of the COI Gene Sequence

**Figure 3:**
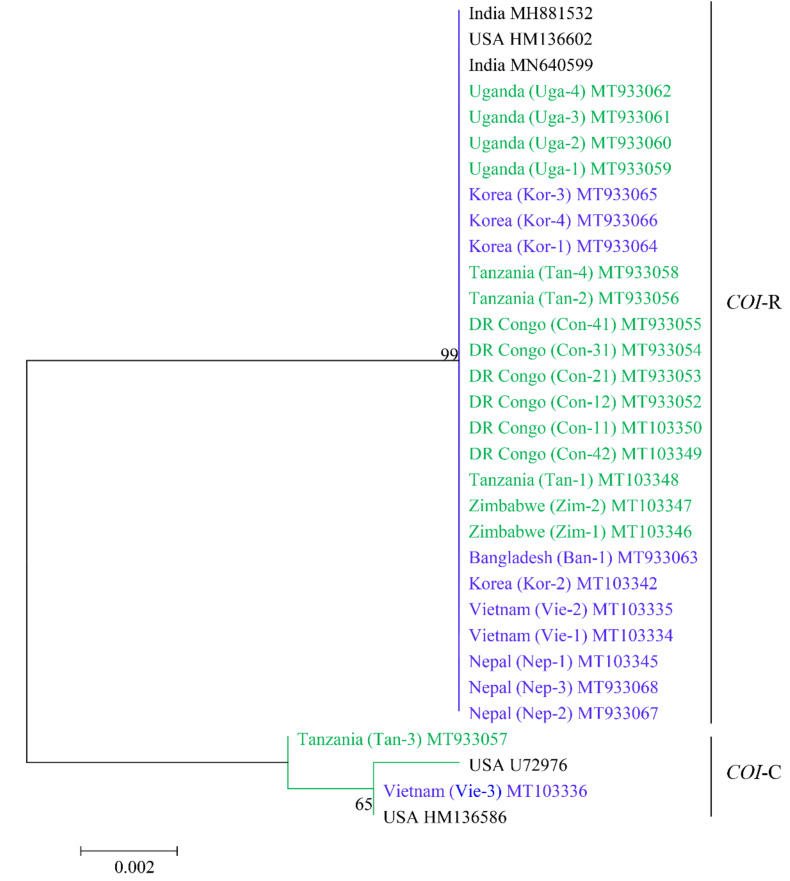
The maximum likelihood phylogenetic tree of the *COI* sequences of *Spodoptera frugiperda* collected from the different African and Asian countries. The color indicates the *COI* sequences from collected samples in this study, and the others are reference sequences obtained from the GenBank database. Hasegawa-Kishnio-Yano HKY850 model and gamma distribution rate of variation among sites were implemented to construct the phylogenetic tree in MEGA6.

The partial sequence (658 bp) of the *COI* gene was determined and phylogenetic relationship was compared with those of previously known sequences from the GenBank database using the maximum likelihood phylogenetic tree (Figure 3). The result showed that 92.6% (25/27) were clustered to the *COI*-rice strain, whereas only 7.4% (Tan-3, Vie-3; 2/27) specimens were clustered to the *COI*-corn strain of FAW. All the specimens were collected from the cornfields. All the *COI*-rice strain sequences (25 specimens) were 100% identical but 98.33–98.48% similar with two sequences of the *COI*-corn strain (two specimens). Two *COI*-corn strain specimens were 99.85% identical (Table A1 and Table A3 in Appendix A).

**Figure 4:**
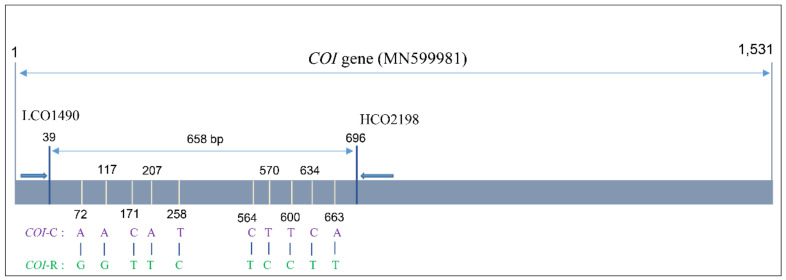
Individual nucleotide differences of the *COI* gene in the corn and the rice strains of *Spodoptera frugiperda*. We used 658 bp from 39 to 696 positions of 1,531 bp of *S. frugiperda COI* gene sequence (MN599981, Korea) from NCBI.

The SNP analysis from the *COI* gene fragment alignment showed that ten nucleotides (mCOI72, mCOI117, mCOI171, mCOI207, mCOI258, mCOI564, mCOI570, mCOI600, mCOI634, and mCOI663) were different between the corn and the rice strains. Based on this comparison, the specimens Tan-3 and Vie-3 belong to the C-strain, and the remaining specimens belong to the R-strain (Figure 4).

### 3.3. Genetic Diversity of Tpi and COI Genes of FAW

The nucleotide sequence variation of the *Tpi* gene was slightly higher in the African specimens (0.23–3.15%) than the Asian specimens (0.23–2.93%), and its variation between Africa and Asia was 0.23–3.38% (Table A2). The numbers of segregating sites, haplotype numbers, haplotype diversity, and nucleotide diversity were higher in Africa than in Asia (Table 2). The nucleotide sequence variation of the *COI* gene was higher in the Asian specimens (1.67%) than in the African ones (1.52%), and its variation between Africa and Asia was 0.15–1.67% (Table A3). The numbers of segregating sites, haplotype diversity, and nucleotide diversity were almost similar between African and Asian specimens (Table 2).

The population genetic study of FAW in Africa and Asia was assessed by Tajima’s neutrality test for the *Tpi* and the *COI* genes (Table 2). The results showed that Tajima’s D was positive and non-significant for the *Tpi* gene in both regions, whereas Tajima’s D is negative but significant for the *COI* gene in both Africa and Asia regions suggesting the recent population expansion.

**Figure 5:**
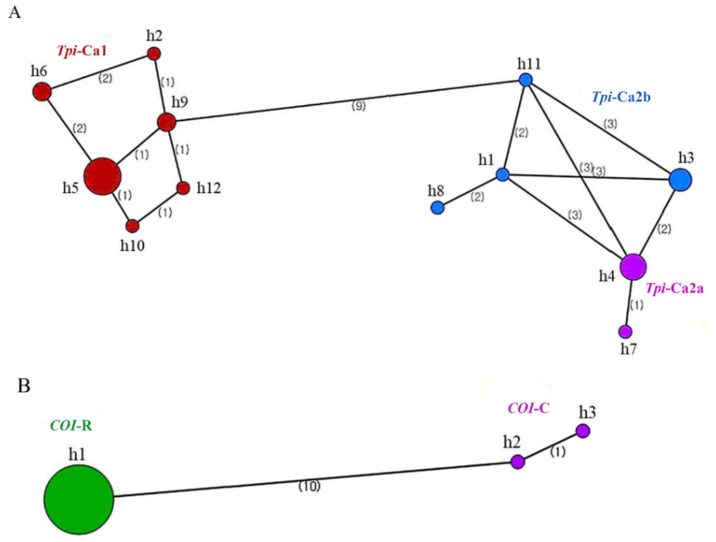
Minimum spanning network of the *Tpi* gene (**A**) and the *COI* gene (**B**) haplotypes of *Spodoptera frugiperda* from different African and Asian countries.

The evolutionary relationship of both *Tpi* and *COI* gene haplotypes from FAW was assessed using the minimum spanning network. In the *Tpi* gene, 12 haplotypes were identified and separated into two distinct groups, *Tpi*-Ca1 and *Tpi*-Ca2, by nine mutational steps (Figure 5A). The *Tpi*-Ca1 consisted of six haplotypes. Among *Tpi*-Ca2, two haplotypes (h4 and h7) belong to the subgroup *Tpi*-Ca2a, whereas four haplotypes (h1, h3, h8, and h11) belong to the subgroup *Tpi*-Ca2b (Table 3). Some identical haplotypes were identified in both Africa and Asia. For example, the h5, which is the most frequent haplotype, was found in two African (Con-42 and Zim-1) and six Asian specimens (Kor-4, Nep-1, Nep-2, Nep-3, Vie-1, and Vie-3). The h6 haplotype was found in one African (Tan-1) and one Asian specimen (Kor-1). The h4 haplotype was found in three African (Con-21, Con-31, and Uga-3) and one Asian specimen (Vie-2).

Only three haplotypes were identified in the *COI* gene, and h1 was differed by ten mutational steps with h2 and h3 (Figure 5B). The h1 haplotype contained 25 specimens from Africa and Asia and belonged to *COI*-R, whereas h2 and h3 haplotypes had a single specimen, Vie-3, and Tan-3, respectively. Both of these haplotypes belonged to the *COI*-C (Table 4). The haplotype analysis of both *Tpi* and *COI* genes indicated that FAW populations invaded in Africa and Asia are genetically diverse at a similar rate. 

## 4. Discussion

In this study, FAW collected from cornfields of eight African and Asian countries were genetically characterized using molecular markers of both the *Tpi* and the *COI* genes. Our *Tpi* gene analysis showed that all the specimens had the *Tpi*-C genotype, whereas the *COI* gene analysis showed that 92.6% had the *COI*-R and 7.4% had the *COI*-C genotypes. Therefore, the hybrid (*Tpi*-C/*COI*-R) was predominant, but the homogenous corn strain (*Tpi*-C/*COI*-C) was a minor genetic group in our survey. This result is similar to previous studies wherein the *Tpi* gene is a predictable molecular marker compared with the *COI* gene for the diagnosis of the FAW strain associated with host plant preference [15,16,17,44]. Another study in Myanmar and Southern China indicated that most of the strain is hybrid (*Tpi*-C/*COI*-R) [45]. It is worth investigating the nuclear *Tpi* gene, a more reliable host strain marker compared with the mitochondrial *COI* marker in invaded populations in Africa and Asia, to prevent further uncertainty on host plant preference analysis.

The genetic variation of both the *Tpi* and the *COI* genes showed that *Tpi* is more diverse compared with *COI*. Moreover, those values were higher for the African specimens than for the Asian specimens. Our data indicated the African populations of FAW are more diversified compared with the Asian ones, especially in the nuclear *Tpi* gene. Nagoshi et al. [6] compared the frequency of both the *Tpi* and the *COI* haplotype combination in the Western Hemisphere and Africa. The homogeneous corn strain (*Tpi*-C/*COI*-C) is predominant in the Western Hemisphere and Western Africa. Both *Tpi*-C/*COI*-C and a hybrid strain (*Tpi*-C/*COI*-R) are similarly distributed in Central Africa, but a hybrid strain predominates in Eastern Africa. Further studies indicated that the hybrid strain predominates in South Africa and India [15,44]. The rice strain (*Tpi*-R/*COI*-R) is found in the Western Hemisphere, but it is rare in Africa [34]. Our data is consistent with previous studies, suggesting that the hybrid strain is predominantly distributed in Africa and Asia while spreading into the east of continents.

Polymorphism of the fourth exon and intron region of the *Tpi* gene is useful for the subgroup identification of FAW [15,44]. Our analysis showed four subgroups of corn strain, such as *Tpi*-Ca1a, *Tpi*-Ca2a, *Tpi*-Ca2b, and *Tpi*-Ca1/*Tpi*-Ca2. Similar profiles are shown in Africa and India, which showed that *Tpi*-Ca1a is the major group, and other subgroups, such as *Tpi*-Ca2a and *Tpi*-Ca2b, are a minor group [15,44]. We found a hybrid (*Tpi*-Ca1/*Tpi*-Ca2) of two subgroups only in one region, Jeju, which is an island located in the southern region of Korea. However, this hybrid was already identified in India at a high frequency [44]. This finding indicates the great potential of further invasion of hybrid strain from India into other Asian countries.

The FAW is a highly polyphagous species that feeds on at least 353 species of plants worldwide [19]. However, the FAW that invaded Africa and Asia mostly prefer corns but not rice and other host plants in the fields, although their major genotype is a hybrid, possessing the nuclear corn strain *Tpi* gene and mitochondrial rice strain *COI* gene [6]. The genetic characteristic of their corn preference is highly associated with the genetic marker of the *Tpi* gene compared with the *COI* gene. Besides, this host plant preference phenotype is not discriminated in the subgroup level, *Tpi*-Ca1, and *Tpi*-Ca2. There are no studies on the relationship between *Tpi* genotype and phenotypic host plant preference. The *Tpi* gene product acts as an essential metabolic enzyme in glycolysis, which catalyzes the reversible reaction of the triose phosphate isomers, dihydroxyacetone phosphate, and D-glyceraldehyde 3-phosphate in the cytosol [46]. The *Tpi* C-strain of FAW may have adaptative mechanisms on the feeding, digestion, and metabolic efficiency of corn plants. It is interesting to study the relationship between the genetic mutation of the *Tpi* gene and metabolic adaptation related to host plant preference.

## 5. Conclusions

In conclusion, the genetic characterization of the *Tpi* and the *COI* genes of African and Asian specimens showed that the *Tpi* gene is a more suitable molecular marker of host plant preference phenotype compared with the *COI* gene. From 2016 to 2020, at least four genetic subgroups of the *Tpi*-corn strain were geographically distributed in Africa and Asia in a similar profile, indicating the limited genetic variation of invaded FAW populations. However, we do not exclude that invaded FAW populations have a great potential to develop genetic adaptations to new environments.

## Figures and Tables

**Table 1:** Specimens’ details of *Spodoptera frugiperda* collected from different African and Asian countries. DR, Democratic Republic.

Regions/ Countries	Locations	Specimen Names	Collection Dates	Insect Stages	Accession Numbers	
					*Tpi*	*COI*
Africa						
DR Congo	Katana, Kabare	Con-11	11/29/2018	Larva	MT894220	MT103350
	Miti, Kabare	Con-12	11/29/2018	Larva	MT894221	MT933052
	Minova, Kalehe	Con-21	11/29/2018	Larva	MT894222	MT933053
	Luvungi, Uvira	Con-31	12/15/2018	Larva	MT894223	MT933054
	Sange, Uvira	Con-41	12/15/2018	Larva	MT894224	MT933055
	Nduba, Walungu	Con-42	12/15/2018	Larva	MT894225	MT103349
Tanzania	Arusha, Tengeru	Tan-1	1/10/2019	Larva	MT894226	MT103348
	Mlali, Morogoro	Tan-2	1/17/2019	Larva	MT894227	MT933056
	Sri, Pwani	Tan-3	1/10/2019	Larva	MT894228	MT933057
	Sua, Morogoro	Tan-4	1/14/2019	Larva	MT894229	MT933058
Uganda	Mbale	Uga-1	1/10/2018	Larva	MT894230	MT933059
	Masindi	Uga-2	10/17/2017	Larva	MT894231	MT933060
	Kole	Uga-3	10/18/2018	Larva	MT894232	MT933061
	Luwero	Uga-4	10/15/2018	Larva	MT894233	MT933062
Zimbabwe	Harare research station, Harare	Zim-1	2/8/2019	Larva	MT894234	MT103346
	Chipinge, Manicaland	Zim-2	2/22/2019	Larva	MT894235	MT103347
Asia						
Bangladesh	Dhaka	Ban-1	8/14/2019	Larva	MT894236	MT933063
Korea	Jeju	Kor-1	9/19/2019	Adult	MT894237	MT933064
	Gyeongsan	Kor-2	8/29/2019	Larva	MT894238	MT103342
	Gyeongsan	Kor-3	6/10/2020	Larva	MT894239	MT933065
	Jeju	Kor-4	6/9/2020	Adult	MT894240	MT933066
Nepal	Bhakundebesi, Kavre	Nep-1	9/24/2019	Larva	MT894241	MT103345
	Khumaltar, Lalitpur	Nep-2	7/30/2019	Larva	MT894242	MT933067
	Khaira, Pyathan	Nep-3	8/6/2019	Larva	MT894243	MT933068
Vietnam	Ninh Binh	Vie-1	9/30/2019	Adult	MT894244	MT103334
	Vinh Phuc	Vie-2	9/30/2019	Adult	MT894245	MT103335
	Hanoi	Vie-3	9/30/2019	Larva	MT894246	MT103336

**Table 2:** Genetic variability analysis of *Tpi* and *COI* gene of *Spodoptera frugiperda* in Africa and Asia.

Genes	Regions	Number of Sequences	Segregating Sites	Haplotypes	Haplotype Diversity	Nucleotide Diversity	Theta/Site	Tajima’s D
*Tpi*	Africa	16	18	10	0.933	0.016911	0.012	1.531362
	Asia	10	14	5	0.667	0.011671	0.011	1.11681
*COI*	Africa	16	10	2	0.125	0.0019	0.005	−2.182611 **
	Asia	11	11	2	0.182	0.00304	0.006	−2.011459 *

* *p* < 0.05, ** *p* < 0.01.

**Table 3:** Specimens and haplotypes of *Spodoptera frugiperda Tpi* gene.

Sn	Speamens	Haplotypes	Strains
1	Con-11	h2	*Tpi*-Ca1
2	Con-42, Kor-4, Nep-1, Nep-2, Nep-3, Vie-1, Vie-3, Zim-1	h5	
3	Kor-2, Tan-1	h6	
4	Tan-3, Zim-2	h9	
5	Uga-1	h10	
6	Uga-4	h12	
7	Con-21, Con-31, Uga-3, Vie-2	h4	*Tpi*-Ca2a
8	Kor-3	h7	
9	Ban-1	h1	*Tpi*-Ca2b
10	Con-12, Con-14, Tan-4	h3	
11	Tan-2	h8	
12	Uga-2	h11	

**Table 4:** Specimens and haplotypes of *Spodoptera frugiperda COI* gene.

Sn	Speamens	Haplotypes	Strains
1	Ban-1, Kor-1, Kor-2, Kor-3, Kor-4, Nep-1, Nep-2, Nep-3, Vie-1, Vie-2, Con-11, Con-12, Con-21, Con-31, Con-41, Con-42, Tan-1, Tan-2, Tan-4, Uga-1, Uga-2, Uga-3, Uga-4, Zim-1, Zim-2	h1	*COI*-R
2	Vie-3	h2	*COI*-C
3	Tan-3	h3	

## Data Availability

The genetic data presented in this study are publicly available on GenBank, and the accession numbers are reported in Table 1.

## Appendix A

**Table A1:** Accession numbers of *Tpi* and *COI* gene sequences of *Spodoptera frugiperda* from different countries of Africa and Asia their identity searched in the GenBank database.

Specimens	Highest Sequence Identity with GenBank Database					
	*Tpi*			*COI*		
	% Identity	Accession Numbers	Countries	% Identity	Accession Numbers	Countries
Con-11	99.77	KT336237	USA	100	MT605970	India
Con-12	99.54	KT336239	USA	100	MT605970	India
Con-21	100	KT336239	USA	100	MT605970	India
Con-31	100	KT336239	USA	100	MT605970	India
Con-41	99.54	KT336239	USA	100	MT605970	India
Con-42	100	KT336236	USA	100	MT605970	India
Tan-1	99.54	KT336236	USA	100	MT605970	India
Tan-2	99.86	KT336239	USA	100	MT605970	India
Tan-3	100	KT336237	USA	99.85	MN541574	India
Tan-4	99.54	KT336239	USA	100	MT605970	India
Uga-1	99.77	KT336236	USA	100	MT605970	India
Uga-2	99.54	KT336229	USA	100	MT605970	India
Uga-3	100	KT336239	USA	100	MT605970	India
Uga-4	99.77	KT336237	USA	100	MT605970	India
Zim-1	100	KT336236	USA	100	MT605970	India
Zim-2	100	KT336237	USA	100	MT605970	India
Ban-1	100	KT336229	USA	100	MT605970	India
Kor-1	97.3	FO681385	France	100	MT605970	India
Kor-2	99.54	KT336236	USA	100	MT605970	India
Kor-3	99.77	KT336239	USA	100	MT605970	India
Kor-4	100	KT336236	USA	100	MT605970	India
Nep-1	100	KT336236	USA	100	MT605970	India
Nep-2	100	KT336236	USA	100	MT605970	India
Nep-3	100	KT336236	USA	100	MT605970	India
Vie-1	100	KT336236	USA	100	MT605970	India
Vie-2	100	KT336239	USA	100	MT605970	India
Vie-3	100	KT336236	USA	100	MN541574	India

**Table A2:** Percentage identity matrix of *Spodoptera frugiperda Tpi* gene analysis from different African and Asian countries.

SN	Specimens	1	2	3	4	5	6	7	8	9	10	11	12	13	14	15	16	17	18	19	20	21	22	23	24	25	26
1	Ban-1																										
2	Kor-1	97.07																									
3	Kor-2	97.30	97.07																								
4	Kor-3	99.10	97.30	97.30																							
5	Kor-4	97.30	97.07	99.55	97.30																						
6	Nep-1	97.30	97.07	99.55	97.30	100.00																					
7	Nep-2	97.30	97.07	99.55	97.30	100.00	100.00																				
8	Nep-3	97.30	97.07	99.55	97.30	100.00	100.00	100.00																			
9	Vie-1	97.30	97.07	99.55	97.30	100.00	100.00	100.00	100.00																		
10	Vie-2	99.32	97.30	97.52	99.77	97.07	97.07	97.07	97.07	97.07																	
11	Vie-3	97.30	97.07	99.55	97.30	100.00	100.00	100.00	100.00	100.00	97.07																
12	Con-11	97.30	97.30	99.55	97.75	99.55	99.55	99.55	99.55	99.55	97.52	99.55															
13	Con-12	99.32	97.07	97.52	99.32	97.07	97.07	97.07	97.07	97.07	99.55	97.07	97.52														
14	Con-21	99.32	97.30	97.52	99.77	97.07	97.07	97.07	97.07	97.07	100.00	97.07	97.52	99.55													
15	Con-31	99.32	97.30	97.52	99.77	97.07	97.07	97.07	97.07	97.07	100.00	97.07	97.52	99.55	100.00												
16	Con-41	99.32	97.07	97.52	99.32	97.07	97.07	97.07	97.07	97.07	99.55	97.07	97.52	100.00	99.55	99.55											
17	Con-42	97.30	97.07	99.55	97.30	100.00	100.00	100.00	100.00	100.00	97.07	100.00	99.55	97.07	97.07	97.07	97.07										
18	Tan-1	97.30	97.07	100.00	97.30	99.55	99.55	99.55	99.55	99.55	97.52	99.55	99.55	97.52	97.52	97.52	97.52	99.55									
19	Tan-2	99.55	96.62	96.85	98.65	96.85	96.85	96.85	96.85	96.85	98.87	96.85	96.85	98.87	98.87	98.87	98.87	96.85	96.85								
20	Tan-3	97.52	97.30	99.32	97.52	99.77	99.77	99.77	99.77	99.77	97.30	99.77	99.77	97.30	97.30	97.30	97.30	99.77	99.32	97.07							
21	Tan-4	99.32	97.07	97.52	99.32	97.07	97.07	97.07	97.07	97.07	99.55	97.07	97.52	100.00	99.55	99.55	100.00	97.07	97.52	98.87	97.30						
22	Uga-1	97.07	96.85	99.32	97.07	99.77	99.77	99.77	99.77	99.77	96.85	99.77	99.32	96.85	96.85	96.85	96.85	99.77	99.32	96.62	99.55	96.85					
23	Uga-2	99.55	97.30	97.75	99.10	97.75	97.75	97.75	97.75	97.75	99.32	97.75	97.75	99.32	99.32	99.32	99.32	97.75	97.75	99.10	97.97	99.32	97.52				
24	Uga-3	99.32	97.30	97.52	99.77	97.07	97.07	97.07	97.07	97.07	100.00	97.07	97.52	99.55	100.00	100.00	99.55	97.07	97.52	98.87	97.30	99.55	96.85	99.32			
25	Uga-4	97.30	97.07	99.10	97.30	99.55	99.55	99.55	99.55	99.55	97.07	99.55	99.55	97.07	97.07	97.07	97.07	99.55	99.10	96.85	99.77	97.07	99.77	97.75	97.07		
26	Zim-1	97.30	97.07	99.55	97.30	100.00	100.00	100.00	100.00	100.00	97.07	100.00	99.55	97.07	97.07	97.07	97.07	100.00	99.55	96.85	99.77	97.07	99.77	97.75	97.07	99.55	
27	Zim-2	97.52	97.30	99.32	97.52	99.77	99.77	99.77	99.77	99.77	97.30	99.77	99.77	97.30	97.30	97.30	97.30	99.77	99.32	97.07	100.00	97.30	99.55	97.97	97.30	99.77	99.77

**Table A3:** Percentage identity matrix of *Spodoptera frugiperda COI* gene analysis from different African and Asian countries.

SN	Specimens	1	2	3	4	5	6	7	8	9	10	11	12	13	14	15	16	17	18	19	20	21	22	23	24	25	26
1	Ban-1																										
2	Kor-1	100																									
3	Kor-2	100	100																								
4	Kor-3	100	100	100																							
5	Kor-4	100	100	100	100																						
6	Nep-1	100	100	100	100	100																					
7	Nep-2	100	100	100	100	100	100																				
8	Nep-3	100	100	100	100	100	100	100																			
9	Vie-1	100	100	100	100	100	100	100	100																		
10	Vie-2	100	100	100	100	100	100	100	100	100																	
11	Vie-3	98.33	98.33	98.33	98.33	98.33	98.33	98.33	98.33	98.33	98.33																
12	Con-11	100	100	100	100	100	100	100	100	100	100	98.33															
13	Con-12	100	100	100	100	100	100	100	100	100	100	98.33	100														
14	Con-21	100	100	100	100	100	100	100	100	100	100	98.33	100	100													
15	Con-31	100	100	100	100	100	100	100	100	100	100	98.33	100	100	100												
16	Con-41	100	100	100	100	100	100	100	100	100	100	98.33	100	100	100	100											
17	Con-42	100	100	100	100	100	100	100	100	100	100	98.33	100	100	100	100	100										
18	Tan-1	100	100	100	100	100	100	100	100	100	100	98.33	100	100	100	100	100	100									
19	Tan-2	100	100	100	100	100	100	100	100	100	100	98.33	100	100	100	100	100	100	100								
20	Tan-3	98.48	98.48	98.48	98.48	98.48	98.48	98.48	98.48	98.48	98.48	99.85	98.48	98.48	98.48	98.48	98.48	98.48	98.48	98.48							
21	Tan-4	100	100	100	100	100	100	100	100	100	100	98.33	100	100	100	100	100	100	100	100	98.48						
22	Uga-1	100	100	100	100	100	100	100	100	100	100	98.33	100	100	100	100	100	100	100	100	98.48	100					
23	Uga-2	100	100	100	100	100	100	100	100	100	100	98.33	100	100	100	100	100	100	100	100	98.48	100	100				
24	Uga-3	100	100	100	100	100	100	100	100	100	100	98.33	100	100	100	100	100	100	100	100	98.48	100	100	100			
25	Uga-4	100	100	100	100	100	100	100	100	100	100	98.33	100	100	100	100	100	100	100	100	98.48	100	100	100	100		
26	Zim-1	100	100	100	100	100	100	100	100	100	100	98.33	100	100	100	100	100	100	100	100	98.48	100	100	100	100	100	
27	Zim-2	100	100	100	100	100	100	100	100	100	100	98.33	100	100	100	100	100	100	100	100	98.48	100	100	100	100	100	100
